# Quality improvement in postnatal care: Findings from two cohorts of women in Sweden

**DOI:** 10.18332/ejm/128737

**Published:** 2020-11-23

**Authors:** Ulrika Öhrn, Helene Parment, Ingegerd Hildingsson

**Affiliations:** 1 Sundsvall Regional Hospital, Sundsvall, Sweden; 2 Department of Women’s and Children’s Health, Uppsala University, Uppsala, Sweden

**Keywords:** cohort study, postnatal care, satisfaction

## Abstract

**INTRODUCTION:**

Postnatal care is an important area of midwifery practice. Changes in the length of postnatal stay, models of postnatal care, and the content of care have influenced women’s satisfaction. The aim of this study was to describe women’s assessment of postnatal care in a Swedish hospital in 2017, and to compare this with women who gave birth in 2006 in the same hospital.

**METHODS:**

A comparative study was conducted of two cohorts of women who gave birth in 2006 and 2017 in a hospital in the middle-north part of Sweden with 1700 annual births. Data were collected by questionnaires, where data from 2017 were compared with data from 2006.

**RESULTS:**

In all, 366 women who gave birth in 2006 and 342 in 2017 responded. There was a reduction in time of discharge in 2017 and more women went home directly from the labour ward and fewer women had their postnatal stay in the hotel ward, compared to postnatal women in 2006. A higher percentage of women were ‘Very satisfied’ with the overall aspects of postnatal care in 2017 compared to women in 2006. The content of postnatal care showed statistically significant improvements over time for the majority of variables studied, but some women reported not receiving information/help with specific postnatal aspects. Multiparous women, women older than 35 years, and women who had had a caesarean section received less information and practical help.

**CONCLUSIONS:**

The study showed an increase in overall satisfaction with postnatal care over time and most areas were improved. Continuous work is therefore needed in order to improve postnatal care and put the women and their families at the centre of care. More research is needed to try new models of care that will increase satisfaction with postnatal care.

## INTRODUCTION

Postnatal care is an important area of midwifery practice. The early postnatal period in the hospital after birth has undergone major changes the last decades, mainly in terms of reduced length of stay^1,2^ but has also become more family focused^1^. Nilsson et al.^1^ described in a metasynthesis of 10 scientific studies, mainly from the Nordic countries, that it is important for parents to be together in the postnatal setting, to have a say about length of stay and to have support available.

Shifts in views about normality has affected the length of hospital stay after birth^2^. Another reason for the reduced length of early postnatal hospital stay might be financial^3^. A Cochrane Review, of 10 randomised controlled trials comprising 4489 women, concluded that early discharge was not associated with adverse effects on breastfeeding or maternal depression^2^. Most studies have found no association between length of postnatal stay and maternal satisfaction but a recent meta-synthesis of 7 papers concluded that women were most likely to be satisfied with early postnatal discharge if they had access to personal and professional support^4^.

In Sweden, healthy women used to stay in hospital for at least one week in the 1970s; nowadays, most women go home directly six hours after birth, without spending time at the postnatal care. Instead, they take care of themselves and their babies with the support of their partner^1^. Similar to many other countries^1-3^, the length of postnatal stay in Sweden has decreased over time. In 1996 the mean length of stay after a vaginal birth was 2.9 days, ten years later (2006) it was 2.2 days, and in 2016 it was 1.8 days^5^. Short stays (0–1 day) or long stays (5 days) were associated with dissatisfaction with postnatal care in a national Swedish survey conducted around 2000^6^. A regional study conducted in 2007–2008 in the middle north part of Sweden found that the majority of women (79%) assessed the length of stay as sufficient^7^. Models of postnatal care have also shifted, from big postnatal wards to more family friendly wards where the partner can stay overnight, as well as hotel wards for uncomplicated cases^7^.

Postnatal care includes health check-ups, breastfeeding initiation, pre- and post-operative care, information, and support^8^. Women’s views about postnatal care seem to be similar regardless of context. Lavender^9^, highlighted in a recent narrative review that an appropriate care provider is fundamental, and the organisation of postnatal care, such as staffing and resources, is crucial for the quality of postnatal care. The most important issues for dissatisfaction with postnatal care raised by women is poor communication, midwife availability, and lack of emotional support^9^. These issues were also discussed in a scientific commentary by Schmied and Bick^10^, who highlighted the importance of support in the transition to parenthood for women and their families.

Women’s satisfaction with postnatal care is generally lower than with antenatal and intrapartum care^6,11^, but studies show that this assessment can fluctuate over time and by the measures used. In a national Swedish sample of 2782 postnatal women surveyed during the year 2000, 34% were ‘Very satisfied’ with the medical aspects of postnatal care, 27% with the emotional aspects, and 35% with the overall assessment of postnatal care6. In addition, a regional population based Swedish survey of 936 women living in the same region as women in the present study showed that 47% were ‘Very satisfied’ with the medical aspects, 33% with the emotional aspects, and 44% with the overall assessment^7^.

Specific factors were identified as contributing to satisfaction with postnatal care, such as length of stay^6,12^, the partners’ option to stay overnight in the postnatal ward^1,6,13-16^, co-care in the neonatal ward^12^, the ability to rest and have privacy^7^, sufficient information and advice^6,7,17^, physical and emotional health issues^7,18^, and the opportunity to talk through the birth with the assisting midwife^6^.

### Context of the postnatal care in the present study

The hospital under study offers a traditional postnatal ward, mainly for women with complicated pregnancies or births. Women in need of breastfeeding support usually stay at the postnatal ward. Healthy mothers and babies can go home directly from the labour ward after six hours and the first paediatric examination. A hotel ward closely situated to the postnatal ward is often used as an in-between place to stay after discharge. The hotel ward is not staffed with midwives, but if the women need support they are instructed to call to the outpatient clinic or the postnatal ward. If the baby is admitted to the neonatal ward, parents have co-care at the neonatal ward. Partners are usually able to stay overnight in the labour ward before going home, in the hotel ward, or, in most cases, in the postnatal ward.

There is also a postnatal outpatient clinic connected to the postnatal ward, where discharged women are followed up after birth. The midwives who work in the postnatal outpatient clinic make daily phone calls to all discharged women, who thereafter come back to the postnatal outpatient clinic with their babies around 48 hours after birth, to give a blood sample for the detection of multiple metabolic disorders (PKU-test). Five to seven days after birth there is a second paediatric examination performed at the clinic.

### Problem area

Previous studies identified several aspects associated with satisfaction with postnatal care. While some aspects seem to become more positive over the years, there is still a need to further investigate factors, whether or not they have improved. The aim of this study was to describe women’s assessment of postnatal care in 2017 in a Swedish hospital, and to compare this with women who gave birth in 2006.

## METHODS

### Design

A comparative study was conducted of two cohorts of women who gave birth in a hospital in the middle north part of Sweden. The hospital has an annual birth rate of around 1700.

### Sample

Women who gave birth during a 6-month period in 2006 and in 2017 were identified from the electronic database at the hospital and were sent a questionnaire that included a letter with information about the study and a pre-paid envelope. In 2006, the questionnaires were only sent to Swedish-speaking women, but were translated into the five most common languages in the area in 2017 (English, Dari, Arabic, Somali, and Tigrinya). A returned questionnaire was considered consent. Four to six weeks after the questionnaire was sent out, a reminding letter with a new questionnaire was sent to non-respondents. Concurrent with the introduction of the postnatal outpatient clinic, efforts were made to improve postnatal care and create a better work environment, based on the results from the 2006 survey^19^. This was done by arranging workshops focusing on establishing a joint vision and value-based care. All members of the staff were involved in the workshops and negotiated that respectful, individualized care should be at focus. Changes in staff’s attitudes were the most important improvement, but some practical changes also occurred. One example was that the outpatient clinic introduced a follow-up program consisting of daily telephone calls to families who either went home after six hours or stayed at the hotel ward. Return visits were organised, prior to discharge, with a specific time twice a day, for consultation with the paediatrician and a midwife. Earlier, the families had to sit in the waiting area for up to two hours, until the paediatrician showed up. During the scheduled time for the re-visit, the families also met with the midwife in charge of the outpatient clinic, and had the opportunity to receive health check-ups and breastfeeding information if needed. For women who needed to stay on the postnatal ward, efforts were made to secure that the partner could stay overnight. Another improvement was the introduction of a structured breastfeeding observation for all women and babies.

### Questionnaire

The questionnaire was previously used in studies performed in 2004 and 2006 at the same hospital^13,14,19^ and the same questionnaire was also used in 2017. Sociodemographic characteristics (age, marital status, country of birth, education level and parity) as well as pregnancy and birth related questions (length of pregnancy, onset of labour, mode of birth, self-reported complications and women’s birth experience) were collected. Questions about the time of discharge from the postnatal ward, experience of length of hospital stay and model of postnatal care were also included in the questionnaire. Moreover, detailed questions about the content of postnatal care (information, practical issues, organizational issues, and women’s experiences of treatment by the staff) and some overall questions about satisfaction with the medical and emotional aspects of care, as well as an overall assessment with postnatal care were collected. Satisfaction with the detailed questions and the overall aspects were assessed on a 5-point Likert scale, with 1 being ‘Very Satisfied’ and 5 ‘Very dissatisfied’. For some of the detailed questions there was also an option to tick the alternative: ‘Did not receive information/practical help’.

In the analysis, the scales were dichotomized into: 1=Very satisfied, and 0=Not very satisfied. The choice of using ‘Very satisfied’ was based on an assumption that it was important to get an evident picture of what really works in order to improve the care^19-22^. A reliability test yielded a Cronbach alpha value of 0.84 for the 2006 sample and 0.86 for the 2017 sample.

### Analysis

The 2017 sample was compared with data from the 2006 survey, using the same questionnaire. Descriptive statistics and chi-squared tests were used to present the participants. Crude and adjusted odds ratios with a 95% confidence interval were calculated for the explanatory variables. The study was approved by the Regional Ethics committee at Umeå University Dnr 2004-134 and 2017-442-31.

## RESULTS

A total of 366 (73%) completed questionnaires from 2006 and 342 from 2017 (40%) were returned. Table 1 shows that the majority of women were 25–35 years old, of Swedish origin, and living with a partner. There were more foreign-born women in 2017 than in 2006 (p=0.000). Around half of the women had a university education in both samples and there were similar proportions of first-time mothers. There was no difference in length of pregnancy or onset of labour, but a statistically significant decrease in emergency caesarean sections in the 2017 sample (p=0.029). The majority self-reported no birth complications, but there was a reduction in women reporting severe bleeding in 2017 (p=0.022).

**Table 1:** Characteristics of study participants in 2017 compared to 2006

*Characteristics*	*2006 (n=366) n (%)*	*2017 (n=342) n (%)*	p*
**Age** (years)			0.053
<25	30 (8.2)	44 (13.3)	
25–35	250 (68.3)	224 (67.7)	
>35	86 (23.5)	63 (19.0)	
**Country of birth**			0.000
Sweden	346 (94.5)	288 (84.2)	
Other	20 (5.5)	54 (15.8)	
**Marital status**			0.526
Living with a partner	355 (97.3)	329 (96.2)	
Not living with a partner	10 (2.7)	13 (3.8)	
**Education level**			0.705
Contemporary/high school	183 (50.1)	161 (48.5)	
College/university	182 (49.9)	171 (51.5)	
**Parity**			0.098
Primiparous	162 (44.3)	173 (50.6)	
Multiparous	204 (55.7)	169 (49.4)	
**Length of pregnancy** (weeks)			0.622
≥37	341 (93.2)	315 (92.1)	
<37	25 (6.8)	27 (7.9)	
**Onset of labour**			0.148
Spontaneously	283 (78.0)	264 (77.4)	
Induction	41 (11.3)	54 (15.8)	
**Mode of birth**			0.045
Vaginal	262 (72.0)	275 (80.9)	
Instrumental vaginal	33 (9.1)	21 (6.2)	
Elective caesarean section	29 (8.0)	21 (6.2)	
Emergency caesarean section	40 (11.0)	23 (6.8)	
**Self-reported complications**			0.014
None	273 (75.4)	275 (81.1)	
Excessive bleeding	28 (7.7)	8 (2.4)	
Large rupture	22 (6.1)	26 (7.7)	
Infection	5 (1.4)	6 (1.8)	
Other	34 (9.4)	24 (7.1)	

* Chi-squared tests were used to detect differences between the years.

Table 2 shows that there were more women in 2017 who chose the option of early discharge within 24 hours as well as within 48 hours, compared to women in 2006, but there was no difference in women’s assessment of the length of postnatal stay. More women in the sample from 2017 chose to go home directly from the labour ward without staying at the postnatal ward or at the hotel ward, and consequently the proportion of women staying at the hotel ward decreased over time.

**Table 2:** Comparison of length of stay and model of postnatal care in 2017 compared to 2006

	*2006 (n=366) n (%)*	*2017 (n=342) n (%)*	*OR (95% CI)*
**Time of discharge** (hours)			
6–24	110 (30.1)	147 (43.6)	2.23 (1.56–3.18)***
24–48	99 (27.0)	96 (28.1)	1.62 (1.10–2.36)*
≥48 (Ref.)	157 (42.9)	94 (27.5)	1.0
**Opinion about length of stay**			
Too short	45 (12.8)	53 (16.1)	1.29 (0.84–1.99)
Sufficient (Ref.)	281 (79.8)	255 (77.3)	1.0
Too long	26 (7.4)	22 (7.4)	0.93 (0.51–1.68)
**Model of postnatal care**^a^			
Home directly from the labour ward	23 (6.3)	66 (19.4)	2.19 (1.30–3.69)**
Traditional postnatal ward (Ref.)	150 (41.8)	196 (57.5)	1.0
Hotel ward	131 (36.7)	61 (17.9)	0.36 (0.24–0.51)***
Postnatal ward and hotel ward	39 (10.9)	18 (5.3)	0.35 (0.19–0.64)**
Co-care neonatal ward	16 (4.5)	39 (11.7)	1.86 (1.00–3.46)*

a Several options available.

* p <0.05

** p<0.01

*** p<0.001.

Table 3 shows the content of postnatal care and the development from 2006 to 2017. For a lot of the studied variables the proportion of women being ‘Very satisfied’ doubled between the years. The exceptions were that women were less likely to report that they were satisfied with the environment, the food and the opportunities for friends and relatives to visit. There was no difference in women’s assessment of breastfeeding information, pelvic exercises, medical check-ups of the woman, possibilities for rest and privacy, and visits from their partner and the baby’s siblings. There were no major changes when adjusting for background variables, birth outcome and time of discharge.

**Table 3:** Satisfaction with the content of early postnatal care in 2017 compared to 2006

	*2006*	*2017*	*OR (95% CI)*	*AOR (95% CI)*^a^
	*(n=366)*	*(n=342)*		
	*n (%)*	*n (%)*		
***Information about birth***				
**Physical changes after birth**				
Very satisfied	37 (10.2)	61 (17.8)	1.91 (1.23–2.97)**	1.62 (1.06–2.58)*
Less than very satisfied (Ref.)	327 (89.8)	281 (82.2)	1.0	1.0
**Emotional changes after birth**				
Very satisfied	24 (6.6)	49 (14.3)	2.36 (1.41–3.94)**	1.95 (1.13–3.36)*
Less than very satisfied (Ref.)	339 (93.4)	293 (85.7)	1.0	1.0
**Breastfeeding**			
Very satisfied	54 (14.9)	67 (19.6)	1.39 (0.94–2.06)	1.12 (0.73–1.71)
Less than very satisfied (Ref.)	309 (84.4)	275 (80.4)	1.0	1.0
**Sexual life after birth**			
Very satisfied	17 (4.7)	46 (13.5)	3.17 (1.78–5.56)***	2.53(1.35–4.73)**
Less than very satisfied (Ref.)	347 (95.3)	296 (86.5)	1.0	1.0
**Pelvic exercise after birth**			
Very satisfied	48 (13.2)	34 (9.9)	0.72 (0.45–1.15)	0.65 (0.39–1.07)
Less than very satisfied (Ref.)	315 (86.8)	308 (90.1)	1.0	1.0
**The baby’s needs**			
Very satisfied	38 (10.4)	69 (20.2)	2.17 (1.41–3.33)***	1.86 (1.18–2.93)**
Less than very satisfied (Ref.)	327 (89.6)	273 (79.8)	1.0	1.0
**Practical instructions about the baby**			
Very satisfied	22 (6.0)	42 (12.3)	2.17 (1.27–3.73)**	2.23 (1.26–3.92)**
Less than very satisfied (Ref.)	342 (94.0)	300 (87.7)	1.0	1.0
**Hands-on breastfeeding**				
Very satisfied	43 (11.8)	65 (19.0)	1.74 (1.15–2.65)**	1.86 (1.20–2.89)**
Less than very satisfied (Ref.)	320 (88.2)	277 (81.0)	1.0	1.0
***Medical check-ups***				
**The woman**				
Very satisfied	61 (16.9)	79 (23.1)	1.47 (1.01–2.13)	1.34 (0.90–1.99)
Less than very satisfied (Ref.)	299 (83.1)	263 (76.9)	1.0	1.0
**The baby**				
Very satisfied	159 (43.7)	193 (56.4)	1.67 (1.24–2.24)**	1.65 (1.20–2.26)**
Less than very satisfied (Ref.)	205 (56.3)	149 (43.6)	1.0	1.0
***Partner’s opportunities***				
**To be involved in the care**				
Very satisfied	58 (16.0)	93 (27.2)	1.95 (1.35–2.82)***	1.99 (1.34–2.95)***
Less than very satisfied (Ref.)	304 (84.0)	249 (72.8)	1.0	1.0
**To stay overnight**				
Very satisfied	115 (31.9)	178 (52.0)	2.32 (1.70–3.15)***	2.52 (1.80–3.51)***
Less than very satisfied (Ref.)	246 (68.1)	164 (48.0)	1.0	1.0
***Satisfaction with staff***				
**Treated nicely**				
Very satisfied	146 (40.4)	194 (56.7)	1.93 (1.43–2.60)***	1.91 (1.39–2.62)***
Less than very satisfied (Ref.)	215 (59.6)	148 (43.3)	1.0	1.0
**Support**				
Very satisfied	110 (30.5)	155 (45.3)	1.89 (1.38–2.57)***	1.74 (1.25–2.41)**
Less than very satisfied (Ref.)	251 (69.5)	187 (54.7)	1.0	1.0
**Encouragement**				
Very satisfied	108 (29.9)	157 (45.9)	1.98 (1.45–2.71)***	1.81 (1.30–2.50)***
Less than very satisfied (Ref.)	253 (69.1)	185 (54.1)	1.0	1.0
**Help with the baby**				
Very satisfied	44 (12.2)	66 (19.3)	1.71 (1.13–2.59)*	1.90 (1.20–2.99)**
Less than very satisfied (Ref.)	316 (87.8)	276 (80.7)	1.0	1.0
**Possibilities to rest and not being disturbed**				
Very satisfied	105 (29.1)	111 (32.5)	1.17 (0.85–1.61)	1.08 (0.77–1.51)
Less than very satisfied (Ref.)	256 (70.9)	231 (67.5)	1.0	1.0
***Satisfaction with the ward/hotel suite***				
**The environment**				
Very satisfied	122 (33.8)	89 (26.0)	0.68 (0.49–0.95)*	0.59 (0.41–0.84)**
Less than very satisfied (Ref.)	239 (66.2)	253 (74.0)	1.0	1.0
**The food**				
Very satisfied	130 (36.0)	46 (13.5)	0.27 (0.18–0.40)***	0.22 (0.14–0.34)***
Less than very satisfied (Ref.)	231 (87.8)	276 (86.5)	1.0	1.0
***Satisfaction with visiting hours***				
**Partner and the baby’s siblings**				
Very satisfied	143 (39.9)	121 (35.4)	0.82 (0.60–1.18)	0.88 (0.63–1.22)
Less than very satisfied (Ref.)	215 (60.1)	221 (64.6)	1.0	1.0
**Visiting possibilities for close friends and relatives**				
Very satisfied	101 (28.1)	42 (12.3)	0.35 (0.24–0.53)***	0.33 (0.22–0.51)***
Less than very satisfied (Ref.)	259 (71.9)	300 (87.7)	1.0	1.0

a AOR: adjusted odds ratio; adjusted for age, country of birth, mode of birth, and time of discharge.

* p <0.05

** p<0.01

*** p<0.001.

A subgroup analysis comparing foreign-born women with women born in Sweden, from the 2017 sample, showed that foreign-born women were significantly more likely to be satisfied with all information-related and practical aspects, the food, the help available and the visiting hours, compared to Swedish-born women. There were no statistically significant differences in variables measuring the treatment by staff, support, or the environment.

### Women not receiving information/practical help

Some women (7–30%) reported they did not receive information about some aspects of postnatal care. In order to identify if there were particular women who reported that they did not receive information or help, we analysed their background characteristics. Multiparous women were more likely to report not receiving information about bodily changes (p=0.026). Multiparous women (p=0.016) and those delivered by a caesarean section (p=0.036) were more likely to report not receiving information about emotional changes (p=0.003). Lack of breastfeeding information was reported by multiparous women (p=0.000) and those who had a caesarean section (p=0.041); these groups of women also reported a lack of information about the baby’s needs (p=0.016 and p=0.010). Women aged 35 years or more, as well as multiparous women, were less likely to receive practical information about how to take care of the baby (p=0.030 vs p=0.000) or hands-on breastfeeding instruction (p=0.003 vs p=0.000), compared to their counterparts (younger women and primiparous women).

Table 4 shows women’s overall assessment of postnatal care. There was an increase in satisfaction from 2006 and 2017 for the medical and emotional aspects, as well as the overall assessment of postnatal care. Primiparous women were more likely to be satisfied with the medical (OR=1.8; 95% CI: 1.25–2.65) and emotional (OR=1.5; 95% CI: 1.01–2.28) aspects of postnatal care, compared to multiparous women. Women delivered by caesarean section were less likely to be satisfied with the emotional aspects of postnatal care (OR=0.3; 95% CI: 0.21–0.56). There were no other differences in background data and satisfaction.

**Table 4:** Comparison of satisfaction with the medical and emotional aspects and an overall assessment of early postnatal care in 2017 compared to 2006

	*2006*	*2017*	*OR (95% CI)*
	*(n=291)*	*(n=344)*	
	*n (%)*	*n (%)*	
**The medical aspects of postnatal care**			
Very satisfied	73 (20.2)	104 (30.8)	1.75 (1.24–2.47)**
Less than very satisfied (Ref.)	288 (79.8)	234 (69.2)	1.0
**The emotional aspects of postnatal care**			
Very satisfied	41 (11.3)	83 (25.0)	2.61 (1.73–3.92)**
Less than very satisfied (Ref.)	321 (88.7)	249 (75.0)	1.0
**Overall assessment of postnatal care**			
Very satisfied	85 (23.8)	117 (35.0)	1.72 (1.23–2.40)**
Less than very satisfied (Ref.)	272 (76.2)	217 (65.0)	1.0

** p<0.01.

## DISCUSSION

One of the findings of this study was that the majority of variables related to the content of postnatal care and overall satisfaction were rated more positively over time. In addition, the study identified areas that need to be improved and subgroups that could favour having more information/help.

### Length of stay

Almost 64% in the present study went home within 48 hours, regardless of mode of birth. This is slightly more compared with data from a Danish population-based study reporting that 59% went home within 50 hours after birth^23^. We do not have the exact time of discharge, but it is unlikely that it deviates from the national statistics^5^. It is, however, important to notice that women who spent their postnatal stay in the hotel ward were discharged from the hospital (administratively), although the hotel ward is situated next to the postnatal ward. The hospital statistics could therefore be misleading; probably more women who lived at a long distance from the hospital used the hotel option and stayed until the blood sample for the metabolic test was taken and then answered the time they left the hospital and not the time of discharge.

We also found that the majority of women assessed the length of stay sufficient, which indicates that the concept of early discharge is the norm and is well established in Sweden. From a clinical perspective, concerns have been raised about declining breastfeeding rates because of early discharge, but research findings have not yet been able to demonstrate any association between early discharge and breastfeeding rates^2,24,25^. Freedom to decide when to go home, however, has been reported as important for postnatal women^17^.

### Model of postnatal care

The results showed a decline in the use of the hotel ward. When the hotel ward was introduced it became popular, being an in-between option for parents to feel secure and to rest together after the birth^14,19^. One explanation for the increase in parents going home directly from the labour ward (usually around six hours after birth) could be the structured hospital’s follow-up program of daily telephone calls and scheduled return visits that was introduced after the workshops.

### Content of care

For most of the variables studied there was an improvement over time. This might be due to the extensive work being done in the clinic between the two periods, in terms of quality improvement, with workshops organised to strengthen the vision and the values of the clinic. The structured way of follow-up after discharge with daily telephone calls and a structured re-visit might also have helped.

### Women who might need more care

Despite the positive development in most of the areas studied, we identified women that might need more attention and information. It seemed important for multiparous women to access information to the same degree as first-time mothers. One explanation could be that staff may assume the experienced mothers have all the information and skills they need. This is not always the case, as shown in a Canadian study of 6421 women. The researchers reported that primiparous and multiparous women perceived inadequate information on the same topics and to the same extent^26^.

Women in the 2017 sample were most satisfied with the partner’s opportunity to stay overnight, medical checkups for the baby, and treatment from staff, with 57–60% reporting as ‘Very satisfied’. The aspect with the lowest ratings were information about pelvic exercises, information about emotional changes following birth, and practical information about how to take care of the baby (14–17% reporting ‘Very satisfied’). Still, it is important to notice the proportions of women reporting that they did not receive information/practical help. Barimani and Vikström^17^ reported, from a focus group study, that parents emphasized the value of consistent advice about breastfeeding, sufficient check-ups, practical help and information about infant care, and reassurance from the staff.

### Overall assessment

Although there was an increase in the proportion of women who reported being ‘Very satisfied’ with their postnatal care from 2006 to 2017, it is important to notice that the proportion of satisfied women was quite similar to that reported in a 2008 regional study that included the same hospital, as well as two smaller hospitals^7^. The smaller hospitals generated higher proportions of satisfaction. The trend of higher satisfaction in this specific hospital under study probably mirrors the attempts to improve the quality of care after the survey in 2006^7^.

In a regional study covering three hospitals^7^, the overall questions of satisfaction were similarly worded as in the present study. Looking only to figures from the same hospital as in the present study, 39% were very satisfied with the medical aspects of postnatal care in 2008, which in the present study (2017 sample) was around 31%. Quite similar proportions assessed the emotional aspects (24%) and the overall assessment (34%) from 2008 to 2017. In the national survey quite similar proportions were also reported; with 26–35% being ‘Very satisfied’^6^. The question remains: is it possible to increase satisfaction with postnatal care or should hospital managers and women be content with this level of satisfaction? We argue that we still have the challenge to continue developing postnatal care – ‘what is, could still be better’^7^.

Several studies have focused on improvement of postnatal care and increased maternal satisfaction, such as midwifery continuity models of care^27^, interventions to support effective communication^17,26,28^, home visits^29-31^, involvement in decisions about leaving the hospital^23^, and, most importantly, a health system that places the woman at the centre of care^9^. Early postnatal care in hospital provides the opportunity to form the foundation for secure parenthood, and attempts to guarantee a safety net should therefore be promoted. However, staff in postnatal wards need to have the time and resources to develop such care.

### Limitations

This study has certain limitations. The observational design makes it impossible to draw any finite conclusions. The fairly low response rate in the 2017 sample is another problem, regardless of the reminding procedure, which might have introduced selection bias. One strength, though, is the translation of the questionnaire for the 2017 sample, allowing non-Swedish speaking women to be included in the study. Another issue is the dichotomization of variables, such as comparing being ‘Very satisfied’ with ‘Less than very satisfied’. However, Carr-Hill^21^, and Brown and Lumley^20^ , suggested that all levels other than being ‘Very satisfied’ should be considered as suboptimal care, and being less than ‘Very satisfied’ indicates areas that could be improved.

## CONCLUSIONS

The study showed an increase in satisfaction with postnatal care over time. Areas of improvement were identified but there are still problems in postnatal care that need to be addressed. Continuous work is therefore needed in order to improve postnatal care and put the women and the families at the centre of care. More research is needed to try new models of care that will increase satisfaction with postnatal care.
